# Influence of Bitumen Type and Asphalt Mixture Composition on Low-Temperature Strength Properties According to Various Test Methods

**DOI:** 10.3390/ma11112118

**Published:** 2018-10-28

**Authors:** Marek Pszczola, Cezary Szydlowski

**Affiliations:** Faculty of Civil and Environmental Engineering, Gdansk University of Technology, 80-233 Gdansk, Poland; cezszydl@pg.edu.pl

**Keywords:** asphalt mixture, low-temperature cracking, tensile strength, strength reserve, flexural strength, uniaxial tension stress test (UTST), Thermal Stress Restrained Specimen Test (TSRST), Bending Beam Test (BBT), Semi-Circular Bending Test (SCB)

## Abstract

In regions with low-temperatures, action transverse cracks can appear in asphalt pavements as a result of thermal stresses that exceed the fracture strength of materials used in asphalt layers. To better understand thermal cracking phenomenon, strength properties of different asphalt mixtures were investigated. Four test methods were used to assess the influence of bitumen type and mixture composition on tensile strength properties of asphalt mixtures: tensile strength was measured using the thermal stress restrained specimen test (TSRST) and the uniaxial tension stress test (UTST), flexural strength was measured using the bending beam test (BBT), and fracture toughness was measured using the semi-circular bending test (SCB). The strength reserve behavior of tested asphalt mixtures was assessed as well. The influence of cooling rate on the strength reserve was investigated and correlations between results from different test methods were also analyzed and discussed. It was observed that the type of bitumen was a factor of crucial importance to low-temperature properties of the tested asphalt concretes. This conclusion was valid for all test methods that were used. It was also observed that the level of cooling rate influenced the strength reserve and, in consequence, resistance to low-temperature cracking. It was concluded that reasonably good correlations were observed between strength results for the UTST, BBT, and SCB test methods.

## 1. Introduction

### 1.1. Background

Many materials used in construction and tested in the laboratory are sensitive to temperature action. Asphalt mixtures belong to materials that extend when they are heated and contract when they are cooled [1]. In cold-climate conditions, asphalt pavements can be subjected to extreme cooling rates that create tensile stresses. The asphalt layer is constrained in the road pavement structure and unable to relieve thermal stresses by internal relaxation. As a result of low-temperature action, thermal tensile stresses increase and when they exceed the fracture strength, low-temperature cracks may appear in the pavement. In the literature, low-temperature cracking is described as a form of thermal cracking that results from a single drop in temperature to an extremely low value observed during severe winters [2,3,4,5,6,7]. The critical cracking temperature may be determined in laboratory using the thermal stress restrained specimen test (TSRST), which was utilized by Monismith et al. [8], Jung and Vinson [9], Pucci [10], and performed according to European standard EN 12697-46 [11]. In spite of many advantages, the TSRST method also has limitations. Failure temperature obtained from the test depends on the cooling rate. Therefore, the results of thermal stress and temperature at fracture should be regarded rather as a comparative measure between asphalt mixtures. The low-temperature properties of asphalt mixtures can be also compared with the results of other methods and indirectly derived from bitumen properties [12]. Epps [13] and Velasquez et al. [14] concluded that the TSRST is recommended for evaluation of thermal behavior of asphalt mixtures. Binder testing alone may not be sufficient to predict the effect of improvements, such as additives and modified bitumen, regarding mixture resistance to thermal stresses. The TSRST provides a relatively simple test method to facilitate the evaluation of mixture resistance to low-temperature cracking. At the same time, fracture temperatures measured directly with TSRST equipment were more conservative (higher) than the predicted fracture temperatures from the indirect tensile test (IDT). The TSRST fracture temperature and strength were slightly different than the calculated IDT fracture strength and temperature. At the same time, a good correlation was observed between the corrected TSRST fracture strength and transverse cracking severity at five MnROAD cells. MnROAD is a pavement test track made up of various research materials and pavements owned and operated by the Minnesota Department of Transportation, working with its partners. According to Bai and Anderson [15], the tensile strength from the indirect tensile test is similar to the tensile strength from the bending beam test and slightly higher than that from direct tensile tests.

One of the methods that describe direct tensile strength of asphalt mixtures at low temperatures is the uniaxial tension stress test (UTST) [16,17]. Tensile strength reserve is defined as the difference between stress curves derived from both the TSRST and the UTST. The maximum value of this reserve is an important parameter helpful in understanding the low-temperature properties of asphalt mixtures in terms of thermal stresses and traffic loads. The research conducted by Grafmuller et al. [18] using the UTST method showed no consistent relationship between the three stages of specimen production: initial type testing in laboratory, production in a mixing plant, and obtaining samples from pavement after paving. It was also observed that the maximum tensile strength reserve reached higher values for specimens compacted in the laboratory. Moreover, the asphalt mixture produced at a mixing plant reached the maximum reserves at higher temperatures.

According to the literature, tensile strength can be measured in the laboratory using different test methods. Buttlar and Rogue [19] developed the indirect tensile test (IDT) to measure tensile strength and creep compliance of asphalt mixtures at low temperatures. Direct tensile strength of asphalt mixture as a function of temperature can be estimated based on binder properties and mix volumetric composition, as proposed by Teltayev and Radovskiy [20]. Falchetto et al. [21], Marasteanu et al. [22], and Velásquez et al. [23] tested asphalt mixture strength at low temperatures using the bending beam rheometer (BBR). These investigations proved simple linear relationships between TSRST critical cracking temperature and strength and the corresponding BBR values. The BBR test equipment was utilized to assess asphalt mixtures based on a simple size-effect theory. For typically sized specimens, the BBR strength results were converted into equivalent tensile strengths of specimens having the same size as TSRST samples [24]. Another method to describe the strength properties of asphalt mixtures at low temperatures is to use flexural strength (bending beam test). According to Reference [25], no clear correlation was found between flexural strength and indirect tensile strength.

Fracture properties of asphalt pavements can be defined on the basis of fracture mechanics theory and can be strictly related to laboratory test results. There are several test methods to assess fracture parameters: bending of single edge notched beams (SENB), bending of semi-circular beams (SCB), and tension of disc-shaped specimens (DC-T) [26]. There were also some trials reported with notched specimens using the TSRST [27]. One of the most suitable and frequently used methods is the bending test of semi-circular specimens (SCB). The basic parameter that defines the strength properties of an asphalt mixture in the SCB test is the fracture toughness *K*_IC_ [28]. For better cracking characterization, more parameters can be assessed, like fracture energy (pre-peak and post-peak), toughness index, and flexibility index [29].

### 1.2. Objectives

The main objective of the paper is to assess the influence of bitumen type and mixture composition on tensile strength properties of asphalt mixtures and evaluate correlations between tensile strength (uniaxial tension stress test), flexural strength (bending beam test), and fracture toughness (semi-circular bending test). The strength reserve behavior of tested asphalt mixtures and the influence of cooling rate on strength reserve were analyzed and discussed as well.

## 2. Materials and Methods

### 2.1. Materials

#### 2.1.1. Bitumens

Four types of bitumen were selected for assessment of low-temperature properties of asphalt mixtures: three neat road bitumens 35/50, 50/70, 70/100, and one polymer Styrene-Butadiene-Styrene (SBS)-modified bitumen 45/80–55. The bitumens came from two different Polish refineries. Standard properties of the bitumens used in this research are shown in Table 1.

#### 2.1.2. Asphalt Mixtures

Laboratory tests were conducted on three types of asphalt mixtures: two asphalt concretes for wearing course layer (AC 11 S for low traffic KR1 ÷ 2, AC 11S for medium traffic KR3 ÷ 4), and one asphalt concrete for the binder course layer (AC 11 W for medium traffic KR3 ÷ 4). All mixes were designed in compliance with the Polish technical guidelines WT-2 2014 [30] and were prepared in the laboratory [31]. The compositions of mixtures and types of bitumen used are presented in Table 2.

### 2.2. Methods

#### 2.2.1. Uniaxial Tension Tests

**Figure 1:**
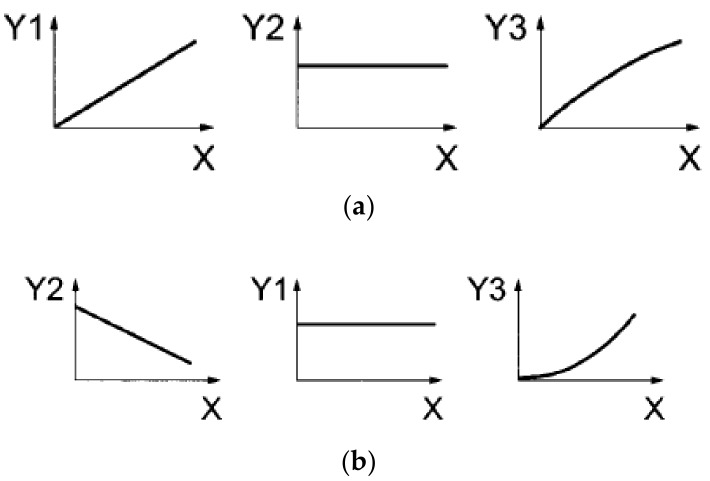
Test principles: (**a**) uniaxial tension stress test (UTST); and (**b**) thermal stress restrained specimen test (TSRST), where: X—time, Y1—strain, Y2—temperature, and Y3—stress [11].

Strength properties of asphalt mixtures at low temperatures were assessed by means of uniaxial tension test methods according to EN 12697-46 standard [11]. Two test methods were used: the uniaxial tension stress test (UTST) and the thermal stress restrained specimen test (TSRST). In the UTST, the specimen is pulled with a constant strain rate at a constant temperature until failure. Results of the UTST are the values of the maximum stress (tensile strength) *β_t_(T)*, and the corresponding tensile failure strain *ε_failure_(T)* at the test temperature *T*. In the TSRST, the specimen, whose length is held constant, is subjected to a decrease in temperature at a constant rate. Due to the prohibited thermal shrinkage, cryogenic (thermal) stress is built up in the specimen. The results of the test are the progression of the cryogenic (thermal) stress over the temperature *σ_cry_(T)* and the failure stress *σ_cry_*, *_failure_(T)* at the failure temperature *T_failure_*. The failure stress is equivalent to strength of the specimen at the failure temperature. The principles of both test methods are shown in Figure 1.

**Figure 2:**
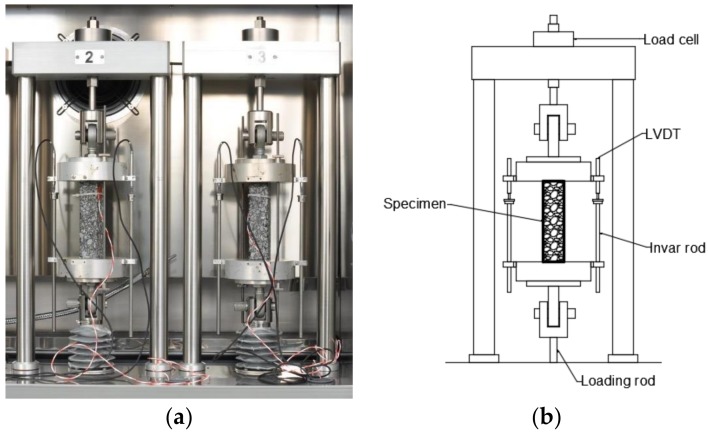
Uniaxial tension stress test (UTST) and thermal stress restrained specimen test (TSRST) setup: (**a**) photograph of specimens during the test; and (**b**) schematic view.

The specimens were tested using TSRST—MULTI Multi Station Thermal Asphalt System servo electric equipment (PAVETEST, Italy). The equipment and test setup used are presented in Figure 2.

Three specimens were tested for each asphalt mixture and test method (UTST or TSRST). The specimens were sawn from larger slabs compacted in laboratory according to EN-12697-33 to obtain the shape of a prismatic beam with dimensions: 40 mm × 40 mm × 160 mm. The specimens were sawn from the central part of the slab with the distance of each specimen to the edge being at least 20 mm.

In the UTST procedure, the specimens were tested at constant test temperatures: −20 °C, −10 °C, +5 °C, and +20 °C. In the case of the asphalt mixture with SBS-polymer modified bitumen, the specimens were also tested at the temperature of −30 °C. The constant strain rate applied in the UTST was Δε = 0.625 ± 0.025%/min, which corresponds to a tension rate of 1 mm/min.

In the TSRST procedure, the specimen is held at a constant length, while temperature is decreased at a uniform rate. The test starts at the temperature of T_0_ = +20 °C. For the standard test method, the cooling rate is set to 10 °C/h. The thermally-induced (cryogenic) stress in the specimen gradually increases as the temperature decreases, until the specimen fractures. The temperature-dependent thermal (cryogenic) stress *σ_cry_*(*T*) is recorded. At lower temperatures the slope of the stress–temperature curve Δ*σ/ΔT* becomes linear (constant), which means the asphalt mixture behaves like an elastic material. The temperature at the tangent point (Tg) is defined by the intersection between the two tangents of the stress-temperature curve: at the aforementioned elastic zone and at the stress relaxation zone, which occurs around the start point of the test at the temperature of +20 °C.

On the basis of the UTST and TSRST results, the tension strength reserve Δ*β_t_*(*T*) for each asphalt mixture was derived. The tensile strength reserve was calculated as the difference between the tensile strength *β_t_*(*T*) (obtained from the UTST as the temperature/tensile strength diagram using a cubic spline function) and the cryogenic (thermal) stress *σ_cry_*(*T*) obtained from the TSRST at the same temperature *T*, using Equation (1):

(1) $$\Delta \beta_{t}\left(T\right)=\beta_{t}\left(T\right)-\sigma_{cry}\left(T\right)$$

where: $\Delta \beta_{t}\left(T\right)$—tensile strength reserve, MPa; $\beta_{t}\left(T\right)$—tensile strength, MPa; and *σ_cry_(T)*—cryogenic (thermal) stress, MPa.

#### 2.2.2. Bending Beam Test (BBT)

The basic procedure of the bending beam test with a constant deflection rate was developed by Judycki [25] and later improved [32,33]. In this article the latest modification is presented. In the test, at least five prismatic specimens (50 mm × 50 mm × 300 mm) were used for every test temperature. Specimens were sawn from plates (300 mm × 300 mm × 50 mm) made of asphalt mixture compacted using standard laboratory roller compactor. The degree of compaction was equal to 99% of Marshall specimen bulk density.

**Figure 3:**
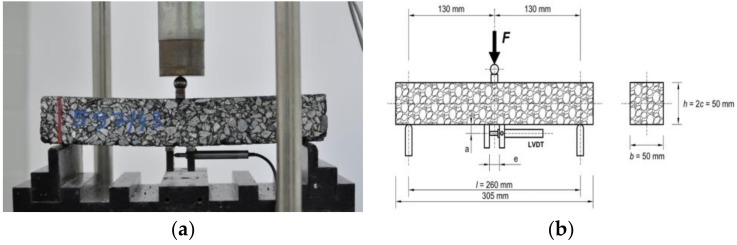
Bending beam test (BBT) with a constant deflection rate setup: (**a**) photograph of the test setup showing the specimen during the test; and (**b**) the schematic view.

The specimen was tested in the mode of three-point bending with a constant deflection rate of 1.25 mm/min. In this test setup, a concentrated load was applied at the midspan of a simply supported beam, measured with two linear variable differential transformers (LVDT’s), which caused deflections that constantly increased in time. One LVDT transducer was positioned horizontally and measured the deformation on the bottom surface of the specimen. The basic temperature set comprised of two temperatures: −20 °C and +10 °C. Before the test, each specimen was subjected to the testing temperature for 12 h. The test setup is presented in Figure 3.

The flexural failure strain at the bottom of the beam subjected to bending was calculated as follows:

(2) $$\varepsilon_{fail}=\frac{p}{e}\cdot \frac{c}{c+a}$$

where: *ε_fail_*—flexural failure strain at the bottom of the specimen (the strain at failure of specimen or at the ultimate stress); *p*—displacement of the LVDT transducer; *e*—the length of the measuring base, mm; *c*—the distance between the center and bottom of the specimen, mm; and *a*—the distance between the bottom of the specimen and the center of the LVDT transducer, mm.

Stress at the bottom of the specimen is calculated using the following equation:

(3) $$\sigma =\frac{M}{W}=\frac{3Fl}{2bh^{2}}$$

where: *σ*—stress at the bottom of the specimen in the center of the length, MPa; *M*—bending moment in the center of the specimen, kNm; *W*—moment of inertia in the cross section, m^3^; *F*—force applied, kN; and *l*, *h*, *b*—specimen dimensions, mm.

The specimen flexural strength was calculated as follows:

(4) $$R_{rz}=\frac{3F_{max}l}{2bh^{2}}$$

where: *R_rz_*—the flexural strength, MPa; and *F_max_*—the force measured at failure of samples or upon reaching the ultimate value, kN.

#### 2.2.3. Semi-Circular Bending Test (SCB)

**Figure 4:**
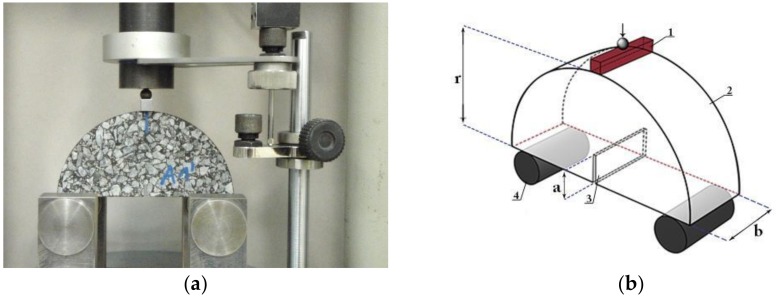
Semi-circular bending test (SCB) with the deflection strain rate setup: (**a**) photograph of the test setup showing the specimen during the test, and (**b**) the schematic view.

Fracture toughness was evaluated using the semi-circular bending test (INFRATEST, Germany) performed according to EN 12697-44 standard [34]. In the test at least four semi-circular specimens were used for every test temperature. Specimens were sawn from gyratory compactor samples (150 mm in diameter and 105 mm in height) made of asphalt mixture. The degree of compaction was equal to 99% of Marshall specimen bulk density. Figure 4 presents specimens and a scheme of the test.

The three tested asphalt mixes were selected with respect to aggregate type (crushed gravel and crushed gneiss) and type of traffic (LT and MT). The original test method described in the EN 12697-44 standard was modified based on a literature review [35,36,37]. The test method was based simply on the determination of the asphalt mixture resistance to fracture *K*_IC_, which was calculated on the basis of the maximum force recorded during the bending of the specimen. The fracture toughness *K*_IC_ was estimated using the following equation:

(5) $$K_{I}=\sigma_{0}Y_{I}\sqrt{\pi a}$$

where: *a*—the notch depth, *σ*_0_ test extreme stress and *Y*_I_—normalized stress intensity factor due to type I fracture.

The extreme bending stress in the specimen was computed using Equation (6), where *F*—the maximum test force, *r*—specimen radius, and *B*—specimen thickness:

(6) $$\sigma_{0}=F/2rB$$

The normalized stress intensity factor was given by the following equation:

(7) $$Y_{I}=4.782-1.219\left(a/r\right)+0.063\exp\left(7.045\left(a/r\right)\right)$$

The major concern is to unify parameters of the experiments: temperature, loading rate, and strain measurement mode [38,39]. In this research the displacement rate was set to 1 mm/min, the same as in the UTST. The specimen and the loading frame during the test were placed in the thermostatic chamber of the press to maintain constant desired test temperatures: −20 °C, −10 °C, 0 °C, +10 °C, and +20 °C. Specimens with a 10 mm notch depth were tested.

#### 2.2.4. Summary of Test Methods and Tested Materials

A recapitulation of the used methods and materials is presented in Table 3.

## 3. Results and Discussion

### 3.1. Results from the Uniaxial Tension Tests and Their Analysis

**Figure 5:**
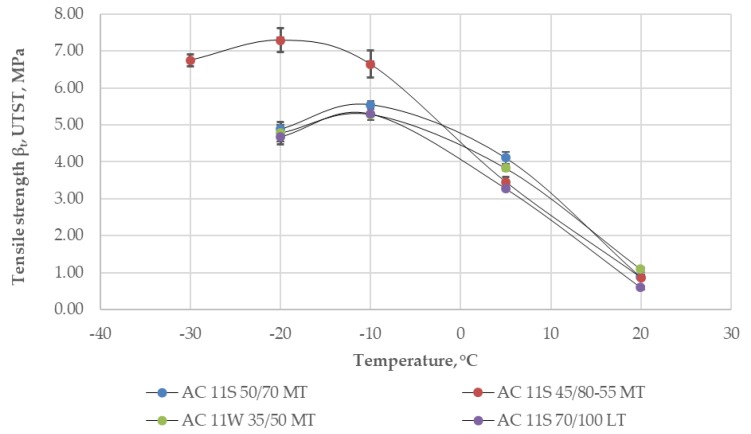
Results of tensile strength from the uniaxial tension stress test (UTST).

Strength properties of the analyzed asphalt mixtures were tested in the uniaxial tension stress test (UTST) at following temperatures: −20 °C, −10 °C, +5 °C, and +20 °C. In the case of AC11S 45/80–55 MT, an additional test temperature was used (−30 °C) due to lack of a visible peak in the test results for standard test conditions. The influence of bitumen type and asphalt mixture on tensile strength *β_t_* is presented in Table 4 and Figure 5.

**Figure 6:**
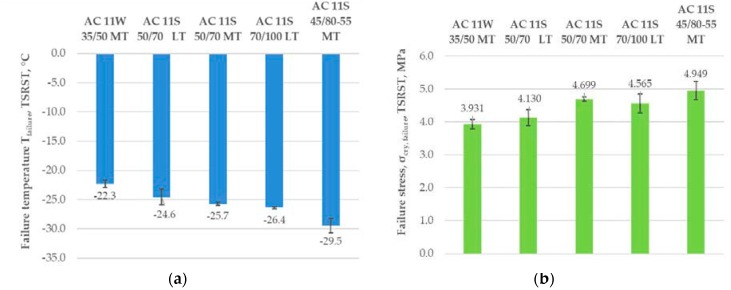
Results of failure temperature and failure stress from the thermal stress restrained specimen test (TSRST): (**a**) failure temperature *T_failure_*, and (**b**) failure stress *σ_cry_*, *_failure_*.

The results show that as the temperature decreased, the tensile strength values increased until they reached their peak values. For all asphalt mixtures that were tested, at the temperature of +20 °C, the tensile strength was almost the same. At the temperature of +5 °C, the asphalt mixtures with harder bitumen types 35/50 and 50/70 showed higher values of tensile strength. Further lowering of the temperature caused a significant increase in tensile strength of the asphalt mixture with SBS-modified bitumen 45/80–55 compared to asphalt mixtures with neat bitumens. According to the literature, low-temperature cracking occurs when thermal tensile stresses exceed the fracture strength of an asphalt pavement layer [1,8,9]. The results presented in Figure 5 show that a polymer-modified binder not only increased the tensile strength of the mixture but also moved the peak value to a lower temperature (peak value changed from about 5.5 MPa at −10 °C for asphalt mixtures with neat bitumens to over 7 MPa at the temperature of −20 °C for the asphalt mixture with SBS-modified bitumen). Thermal tensile stresses (cryogenic stresses according to EN 12697-46) were tested using the thermal stress restrained specimen test (TSRST) and the results are presented in Figure 6.

As shown in Figure 6, the type of bitumen is a factor of crucial importance to low-temperature properties of the tested asphalt mixtures. The best low-temperature properties in the TSRST (the lowest value of failure temperature and the highest value of failure stress) were obtained for the asphalt concrete with SBS-modified bitumen 45/80–55. In contrast, the worst properties (higher value of failure temperature and lower value of failure stress) were obtained for the asphalt concrete with the hardest neat bitumen 35/50. Comparison of asphalt mixture types did not reveal important differences between results for the asphalt concrete AC 11S 50/70 LT designed for light traffic (LT) with a binder content of 5.8% by mass and for the AC 11S 50/70 MT designed for medium traffic (MT) with a binder content of 5.6% by mass.

**Figure 7:**
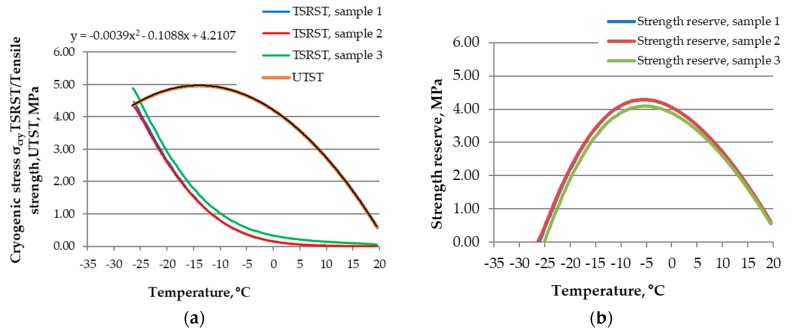
An example of the tensile strength reserve analysis: (**a**) the TSRST and UTST results; and (**b**) a graphical presentation of the tensile strength reserve results.

The UTST and TSRST test results served as a basis for calculation of tensile strength reserve. The calculations were conducted using Equation (1) according to EN 12697-46 [11]. An example of tensile strength reserve analysis is presented in Figure 7. Figure 7a shows the results of the tensile strength (UTST) and the cryogenic stress (TSRST) versus temperature, measured for the standard cooling rate 10 °C/h used in the TSRST procedure. Figure 7b presents the diagrams that are the final result of tensile strength reserve calculations.

**Figure 8:**
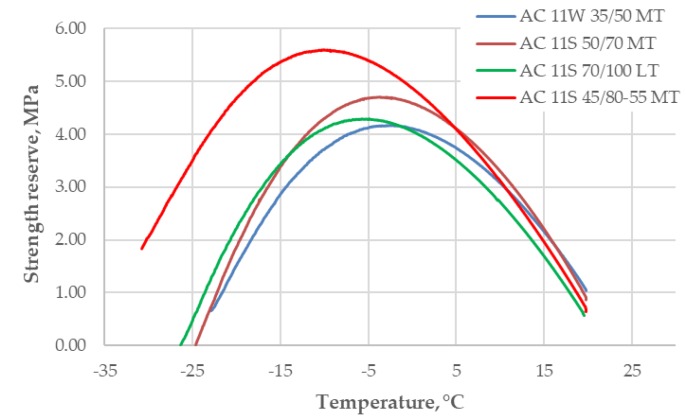
Results of strength reserve calculations.

The results of the strength reserve calculations for all asphalt mixtures are presented in Figure 8.

**Figure 9:**
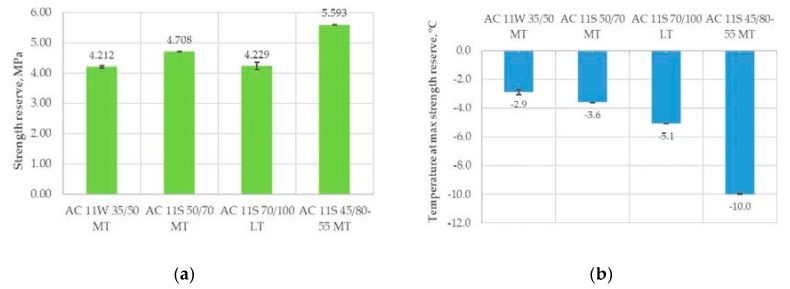
Influence of asphalt mixture and bitumen type on strength reserve results: (**a**) maximum strength reserve results; and (**b**) the temperature at the maximum value of strength reserve.

The interpretation of the results is that the asphalt mixtures with a higher value of strength reserve have a better resistance to low-temperature cracking. There are two indicators of this resistance. One is the maximum value of strength reserve and the second is the temperature at the maximum value of strength reserve. Both are presented in Figure 9. The lower the calculated values of temperature, the better the resistance to low-temperature cracking.

The results of the analysis indicated that the best low-temperature properties were obtained for the asphalt mixture with an SBS-polymer modified bitumen 45/80–55. The results of temperature at the maximum value of strength reserve were particularly clear. The least favorable results were obtained for the asphalt mixture with 35/50 neat bitumen.

**Figure 10:**
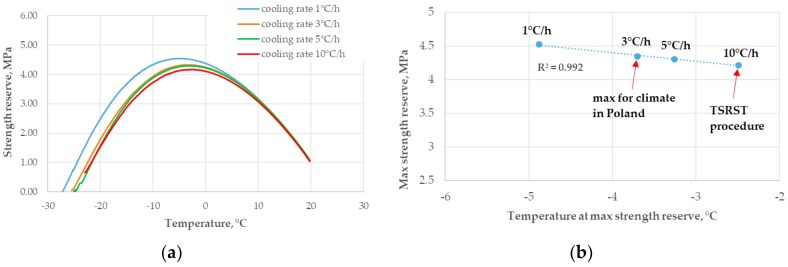
Influence of cooling rate on tensile strength reserve: (**a**) strength reserve for different cooling rates; and (**b**) maximum strength reserve values versus temperature at the maximum strength reserve.

Another important factor that influences the TSRST results and, indirectly, the tensile strength reserve analysis, is the cooling rate. According to the literature [40,41,42], the cooling rate significantly affects the experimental measurements in the TSRST method. The real field-observed cooling rates reported in the literature vary from 0.5 °C/h to 3 °C/h [43]. The cooling rate analysis was performed for the asphalt mixture AC 11W 35/50 MT using four different cooling rates: 1 °C/h, 3 °C/h, 5 °C/h, and 10 °C/h (the latter being the standard in the TSRST procedure). The influence of cooling rate on tensile strength reserve is presented in Figure 10.

It is visible in Figure 10 that with an increase in the cooling rate, the maximum values of strength reserve decreased and the calculated temperature increased. The difference between the observed temperature at the maximum value of strength reserve for a cooling rate of 3 °C/h (that was maximum for actual field conditions) and for cooling rate of 10 °C/h (which was used in the TSRST procedure) is important. For the same asphalt mixture, the level of cooling rate influenced the assessment of resistance to low-temperature cracking. For extreme field conditions (cooling rate 3 °C/h) the strength reserve was higher and temperature at that strength reserve was lower than for the TSRST procedure (cooling rate 10 °C/h).

### 3.2. Results from the Bending Beam Test (BBT) and Their Analysis

The results of flexural strength and flexural failure strain from the bending beam test, sorted according to mixture type and test temperature, are presented in Table 5.

The results presented in Table 5 indicate that for asphalt mixtures that were tested in the BBT at the temperature of −20 °C, the highest value of flexural strength was obtained for the mixture with SBS-polymer modified bitumen 45/80–55. The influence of SBS-polymer modification on the strength properties during bending at the temperature of +10 °C turned out to be less important. Another trend was observed for flexural failure strain values, which are an indicator of elastic properties of the asphalt mixture as well. In that case, better elastic properties were observed for the mixture with SBS-polymer modified bitumen at both test temperatures: −20 °C and +10 °C.

### 3.3. Results of Fracture Toughness from the Semi-Circular Bending Test (SCB) and their Analysis

**Figure 11:**
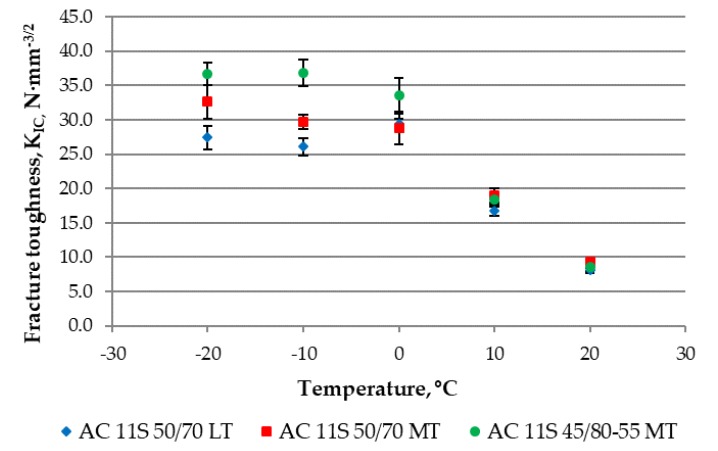
Results of fracture toughness from the semi-circular bending test (SCB).

The results of fracture toughness for different mixture types and temperatures are presented in Table 6 and Figure 11.

SCB test results showed that fracture toughness at intermediate temperatures (+10 °C and +20 °C) does not depend on bitumen type or aggregate type. More visible differences could be seen at low temperatures. The highest fracture strength characterized the asphalt concrete with SBS-modified bitumen. The asphalt concrete with crushed gravel had lower cracking resistance at low temperatures than the asphalt mixture with crushed gneiss. It could be noted as well that fracture toughness for every tested asphalt concrete increased with a decrease in temperature from +20 °C to 0 °C and remained almost constant during a further decrease in temperature from 0 °C to −20 °C.

### 3.4. Correlations between Strength Results Obtained from Different Methods

**Figure 12:**
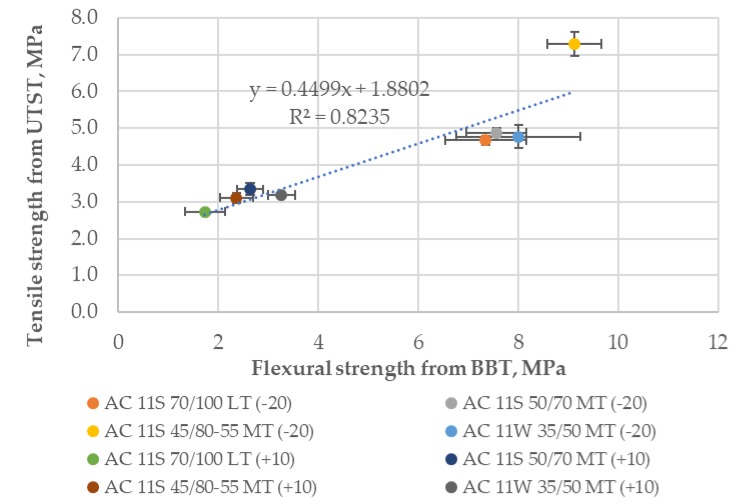
Tensile strength from the UTST versus flexural strength from the BBT.

Three test methods were used to describe the strength properties of the tested asphalt mixtures. The setup of each test method was different, therefore it may be advantageous to analyze whether any correlations between the results exist. Comparison between tensile strength results from the UTST and flexural strength results from the BBT at temperatures of −20 °C and +10 °C is presented in Figure 12. As shown in the plot, the R^2^ value calculated for a linear trend and all the results equals 0.82.

**Figure 13:**
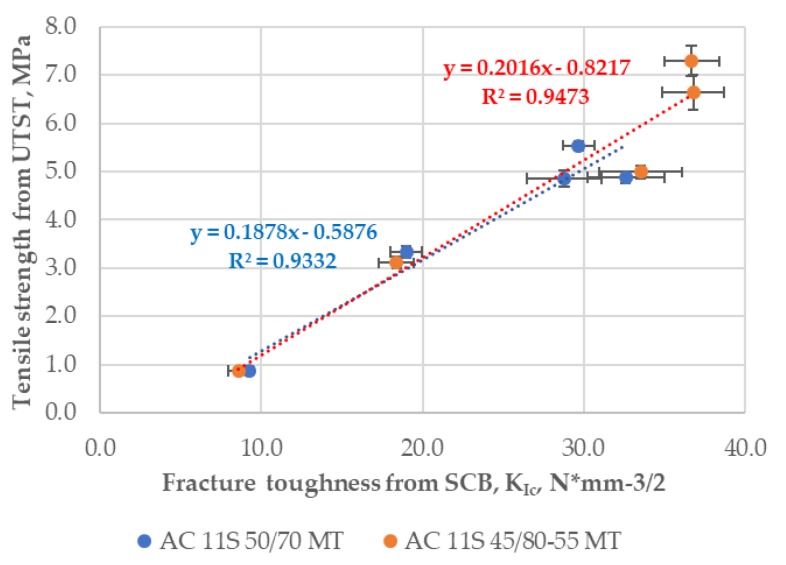
Tensile strength from the UTST versus fracture toughness *K*_IC_ from the SCB test.

The second comparison was analyzed for tensile strength results from the UTST and the SCB fracture toughness for asphalt mixture AC 11S with neat bitumen 50/70 and SBS-polymer modified bitumen 45/80–55 across all the test temperatures from −20 °C to +20 °C. The correlation results are presented in Figure 13. It is noteworthy that the R^2^ value calculated for a linear trend and the results for the asphalt mixture with neat bitumen 50/70 was equal to 0.93, and for the asphalt mixture with SBS-modified bitumen 45/80–55, it was equal to almost 0.95.

**Figure 14:**
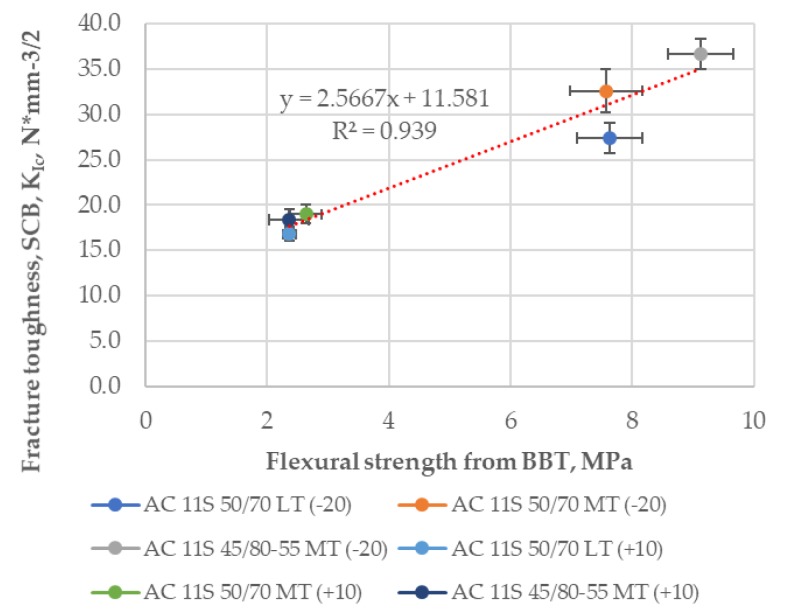
Fracture toughness, *K*_IC_ from the SCB versus flexural strength from the BBT.

The comparison between the SCB fracture toughness results and flexural strength results from the BBT at temperatures of −20 °C and +10 °C is presented in Figure 14. A good correlation can be observed as well. The R^2^ value for a linear trend considering all the results was 0.94.

To summarize the correlation analysis that was conducted, for all test methods—the UTST, BBT, and SCB procedures—reasonably good correlations were observed between the obtained strength results.

## 4. Summary and Conclusions

This work presents the study of low-temperature strength properties of different asphalt mixtures. The influence of bitumen type and mixture composition on tensile strength (UTST), flexural strength (BBT), and fracture toughness (SCB) was analyzed and discussed. The strength reserve behavior of asphalt mixtures and the influence of cooling rate on the strength reserve were investigated as well. Based on the test results and analysis, the following conclusions can be drawn:

1. The type of bitumen is a factor of crucial importance in assessment of low-temperature properties of the tested asphalt mixtures. The results from the UTST show that a polymer-modified binder not only increased the tensile strength of the mixture, but also improved the peak value from about 5.5 MPa at −10 °C for asphalt mixtures with neat bitumens to over 7 MPa at a temperature of −20 °C for the asphalt mixture with SBS-modified bitumen.
2. The best low-temperature properties in the TSRST (the lowest value of failure temperature and the highest value of failure stress) were obtained for the asphalt concrete with SBS-modified bitumen 45/80–55. Other tests—the BBT and the SCB—also proved a better resistance of the asphalt mixture with SBS-modified bitumen to low-temperature cracking.
3. The results of the strength reserve indicated that the best low-temperature properties were obtained for the asphalt mixture with SBS-polymer modified bitumen 45/80–55. The lowest values were obtained for the asphalt mixture with 35/50 neat bitumen.
4. During the assessment of various asphalt mixture types, no important differences were observed between the TSRST results for the asphalt concrete designed for light traffic (LT) with a binder content of 5.8% by mass and for the asphalt concrete for medium traffic (MT) with a binder content of 5.6% by mass.
5. The type of aggregate did influence the fracture toughness of the tested mixes. Asphalt concrete for low traffic with crushed gravel had a lower fracture toughness than the asphalt concrete for medium traffic with crushed gneiss.
6. After the cooling rate investigation, it was observed that the temperature at the maximum value of strength reserve for the cooling rate of 3 °C/h (that is the highest rate under real field conditions) was lower than for the cooling rate of 10 °C/h (which was used in the TSRST procedure).
7. Analysis of correlation between strength results obtained from the UTST, BBT, and SCB indicates reasonably good correlations: 0.82 (UTST versus BBT), 0.94 (UTST versus SCB), and 0.94 (SCB versus BBT).
8. The main limitation of the study was related to the methodology of test methods. The UTST and TSRST tests were conducted according to EN 12697-46 standard. In the UTST, the specimen was pulled with a constant strain rate at a constant temperature until failure. The influence of strain rate on tensile strength of asphalt mixtures at low temperatures will be the purpose of further research.

## Figures and Tables

**Table 1:** Properties of bitumens.

Property		Type of Bitumen			
		35/50	50/70	70/100	45/80–55
Penetration at 25 °C, 0.1 mm, acc. to PN-EN 1426	Original	45	54	81	60
	RTFOT *	28	40	48	40
R&B Temperature, °C, acc. to PN-EN 1427	Original	53.0	50.8	47.8	68.6
	RTFOT	57.8	57.8	53.4	67.4
Performance Grade, acc. to AASHTO M 320		70–16	64–22	58–22	70–22
Fraass Breaking Point Temperature, °C, acc. to PN-EN 12593	Original	−6	−14	−16	−16
	RTFOT	−3	−12	−10	−15

* RTFOT—Rolling Thin-Film Oven Test

**Table 2:** Properties of asphalt mixtures.

Property	Types of Mixtures		
Asphalt mixture	AC 11 S KR1 ÷ 2	AC 11 S KR3 ÷ 4	AC 11 W KR3 ÷ 4
Type of layer	wearing course	wearing course	binder course
Type of traffic	low traffic (LT)	medium traffic (MT)	medium traffic (MT)
Bitumen type	50/70	50/70	35/50
	70/100	45/80–55	
Binder content (% by mass)	5.8	5.6	5.6
Aggregate type	crushed gravel	crushed gneiss/granite	crushed gneiss
Filler type	limestone	limestone	limestone
Sieve size (mm)	% Passing (by mass)		
16	100	100	100
11.2	97	98	98
8	83	77	83
5.6	71	62	65
4	60	52	54
2	40	39	43
0.125	11	11	12
0.063	8	7.2	7.4

**Table 3:** Summary of test methods and tested materials.

Asphalt Mixture and Bitumen Type	UTST	TSRST	BBM	SCB
AC 11S 70/100 LT	X	X	X	-
AC 11S 50/70 LT	-	X	X	X
AC 11S 50/70 MT	X	X	X	X
AC 11S 45/80/55 MT	X	X	X	X
AC 11W 35/50 MT	X	X	X	-

**Table 4:** Results of tensile strength *β_t_* from the uniaxial tension stress test (UTST).

Asphalt Mixture and Bitumen Type		Tensile Strength *β_t_*, MPa				
		−30 °C	−20 °C	−10 °C	+5 °C	+20 °C
AC 11W 35/50 MT	mean value	not tested	4.773	5.277	3.815	1.080
	st. deviation		0.312	0.022	0.064	0.049
	CV, %		6.5	0.4	1.7	4.5
AC 11S 50/70 MT	mean value	not tested	4.881	5.538	4.097	0.871
	st. deviation		0.120	0.098	0.162	0.075
	CV, %		2.5	1.8	3.9	8.6
AC 11S 70/100 LT	mean value	not tested	4.675	5.290	3.269	0.589
	st. deviation		0.122	0.152	0.030	0.043
	CV, %		2.6	2.9	0.91	7.2
AC 11S 45/80–55 MT	mean value	6.747	7.292	6.642	3.457	0.868
	st. deviation	0.154	0.316	0.367	0.135	0.033
	CV, %	2.3	4.3	5.5	3.9	3.8

**Table 5:** Results of flexural strength from the bending beam test (BBT).

Asphalt Mixture and Bitumen Type		Flexural Strength, MPa		Flexural Failure Strain, ‰	
		−20 °C	+10 °C	−20 °C	+10 °C
AC 11W 35/50 MT	mean value	8.01	3.27	0.91	9.13
	st. deviation	1.24	0.27	0.09	0.73
	CV, %	15.5	8.4	9.8	8.0
AC 11S 50/70 MT	mean value	7.57	2.64	0.89	13.12
	st. deviation	0.59	0.26	0.21	2.12
	CV, %	7.9	10.0	24.1	16.2
AC 11S 70/100 LT	mean value	7.35	1.74	0.88	22.07
	st. deviation	0.81	0.41	0.14	6.23
	CV, %	11.0	23.4	16.0	28.2
AC 11S 45/80–55 MT	mean value	9.13	2.37	1.19	30.40
	st. deviation	0.54	0.33	0.21	5.60
	CV, %	5.9	13.7	17.8	18.4

**Table 6:** Results of fracture toughness *K*_IC_ from semi-circular bending test (SCB).

Asphalt Mixture and Bitumen Type		Fracture Toughness *K*_IC_, N·mm^−3/2^				
		−20 °C	−10 °C	0 °C	+10 °C	+20 °C
AC 11S 50/70 LT	mean value	27.4	26.1	29.4	16.8	8.1
	st. deviation	1.7	1.3	0.6	0.8	0.4
	CV, %	6.4	4.9	2.8	4.9	4.5
AC 11S 50/70 MT	mean value	32.6	29.7	28.8	19.0	9.3
	st. deviation	2.4	1.0	2.3	1.0	0.2
	CV, %	7.4	3.4	8.1	5.4	2.0
AC 11S 45/80–55 MT	mean value	36.7	36.8	33.5	18.4	8.6
	st. deviation	1.7	1.9	2.6	1.1	0.6
	CV, %	4.5	5.2	7.7	5.7	6.8
